# Exploring the information quality of mobile government services: a literature review

**DOI:** 10.7717/peerj-cs.1028

**Published:** 2022-07-28

**Authors:** Abdulla Jaafar Desmal, Mohd Khalit Othman, Suraya Hamid, Ali Zolait

**Affiliations:** 1 Faculty of Computer Science and Information Technology, Universiti Malaya, Kuala Lumpur, Malaysia; 2 College of Information Technology, University of Bahrain, Sakheer, Bahrain

**Keywords:** Mobile government, E-service quality, Online information quality, Information quality

## Abstract

This article aims to investigate the constructs that can be used to evaluate the information quality (IQ) of mobile government services. The dimension of IQ is one of the fundamental constructs that assesses the extent of information based on its accuracy, usefulness, and timeliness. Based on the review of previous studies, there is a lack of studies related to mGovernment service quality. It is not practical to measure the service quality of mGovernment by using other measurement scales such as e-service, e-commerce, or e-government. Therefore, it is necessary to understand each dimension that guides constructing a comprehensive framework to measure service quality at mGovernment. The constructs of information quality were extracted from previous literature in mobile government, mobile application service, and e-government to understand the development stages, structure, and unique features—this guide to conduct the systematic literature review to clarify the constructs belonging to the evaluation of information quality. The present article identified six constructs—understandability, timeliness, accuracy, completeness, availability, and usefulness—to measure the information quality of mobile government service. There is limited literature on mGovernment information service quality. With the development of government services on mobile devices, it is necessary to measure information quality at mGovernment service channel to understand users’ expectations. The mGovernment service provider benefits from measuring the service quality by improving the strategy and criteria of information at mGovernment portal. In addition, the end-users expect to perform the service with the best quality level of the information supplied and displayed on mGovernment platform.

## Introduction

The development of information and communications technology has influenced the process of online services. Users of mobile devices are increasing worldwide (Bindu, Sankar & Kumar, 2019; Yang, Elisa & Eliot, 2018). The popularity of smart devices is considered an opportunity to communicate faster and more flexibly (Ahmad & Khalid, 2017; Yang et al., 2018). Users typically use smart devices to access online services and information. This development has changed the routine of interaction between public and government agencies, encouraging governments worldwide to create a way to deliver services through smart devices. Mobile government service (mGovernment, mGov) is a technology for providing government services by using mobile applications to allow the public to interact with government agencies and perform services using smart devices connected *via* a wireless internet connection (Almarashdeh & Alsmadi, 2017; Desmal et al., 2019; Li et al., 2018b). Countries worldwide have improved their mGovernment portals by transmitting traditional and e-services into a mobile portal. Services delivered through the mGovernment portal can be in health, education, business, industries, police, communications, and utilities (Bindu, Sankar & Kumar, 2019; Yang, Elisa & Eliot, 2018). Citizens use the mGovernment portal to get highly professional, best quality services compared with other forms of services (Taamallah, Khemaja & Faiz, 2019).

However, little research in the area of mGovernment service quality discusses service quality in general (Chanana, Agrawal & Punia, 2016), and other studies propose a model to be used for the evaluation of such services by mGovernment (Al-Hubaishi, Ahmad & Hussain, 2017; Shareef et al., 2014). Among these studies, the analysis of quality dimensions that can be used to measure the services of mGovernment is weak, leading the current study to pick one of the main important dimensions of service quality: information quality (IQ). Petter, DeLone & McLean (2013) defined the concept of IQ in an online context as “users’ perception of the quality of information presented on a web site”, while Wang & Lin (2012) defined IQ as “the ability of the system to convey the intended meaning of information”. Previous studies analyzed IQ with its constructs according to the features of the website, but when the service is delivered through mobile devices, the constructs must meet the unique features of such devices. Using other measurement scales in the context of mGovernment service leads to difficulties and inaccurate results.

However, based on previous discussions, the present study aims to analyze the dimension of IQ of mGovernment service quality. The government agencies can use the IQ proposed model of the present study as a service provider to evaluate the services delivered to users, which enables government agencies to understand the users’ satisfaction, expectations and needs toward IQ from mGovernment services. The other important dimensions of mGovernment service quality will be investigated by the authors of the present research in future studies.

## Literature review

### Mobile government

Mobile Government (mGovernment) refers to the use of portable smart devices that are connected to government agencies by a wireless internet connection to deliver the services to the public anytime and anywhere. It is in the form of a mobile application that is used by mobile devices and smart devices (Jaafar Desmal et al., 2019; Jaafar Mohamed et al., 2019). Mandari, Chong & Wye (2017) argue that the mGovernment is considered an extension of electronic government. Services provided by mGovernment can be in the form of Government To Government (G2G), Government To Business (G2B), Government To Citizen (G2C), and Government To Employees (G2E) (Akram et al., 2019; Haneem et al., 2019). The service of mGovernment has attracted the attention of researchers concerning user satisfaction and investigation of adoption factors (Haneem et al., 2019; Theodosiou et al., 2019), studying the challenges faced by countries (Doheim, Farag & Badawi, 2019; Taamallah, Khemaja & Faiz, 2019), while there was lack of investigation of quality evaluation in the context of mGovernment (Al-Hubaishi, Ahmad & Hussain, 2017; Shareef et al., 2014). Some of the unique features of mGovernment services are portability, personalization, and location (Desmal et al., 2019; Ostovare & Shahraki, 2019). The wide acceptance of smart devices has encouraged government agencies to provide more services as mobile applications that enable the public to perform the task anytime and anywhere. When comparing mGovernment and eGovernment, the service-based mobile application is more flexible and efficient to access the services and information. In terms of interaction with government agencies, mGovernment can conduct different interaction with public such as video, audio, and chat.

### Service quality

Quality is defined as the totality of functions, characteristics of behaviors of a good or service. To measure the quality, it is essential to consider evaluating all quality constructs of features, functions, or behaviors. The term “service” is understood to mean any activity of benefit provided by one party to another. Therefore, combining the terms “service” and “quality” leads to identifying the concept of service quality as fulfilling the expectations that the client has about the service and how well the customer is satisfied. A study by Zeithaml, Berry & Parasuraman (1996) defined the concept of service quality as an “overall judgment similar to attitude towards the service and generally accepted as an antecedent of overall customer satisfaction”, while it is defined in a study by Parasuraman, Zeithaml & Berry (1988) as the “ability of the organization to meet or exceed customer expectations”. In an offline environment, previous researchers have proposed different service quality models to evaluate the quality of services, such as the model of SERVQUAL, which is proposed by Parasuraman, Zeithaml & Berry (1988) and consists of ten quality dimensions: reliability, responsiveness, competence, access, courtesy, communication, credibility, security, understandability, and tangible. The model of SERVQUAL became popular, but it cannot be applied to all types of services (Khan, Lima & Mahmud, 2018). Cronin & Taylor (1992) revised the SERVQUAL model and proposed a new quality model called SERVPERF that uses performance to measure the quality of service, while the model of SERVQUAL was proposed according to the model of the expectations and perception of the customer. However, previous researchers in various fields proposed models to measure the quality of service.

In an online environment, Parasuraman, Zeithaml & Malhotra (2005) proposed a service quality model called E-S-QUAL that can be applied in the electronic service environment, consisting of four main dimensions: efficiency, fulfillment, system availability, and privacy. Other researchers proposed different e-service quality models; for example, Liu & Arnett (2000) proposed e-Commerce SQ, consisting of four main dimensions: information and service quality, system use, playfulness, and system design quality. The development of technology and electronic services has resulted in forms of electronic services such as e-commerce, e-services, and eGovernment. Each of these categories has service quality models proposed by researchers. However, in mobile services, the mGovernment service has little research on service quality, and the current study aims to focus more on the details of IQ as a part of measuring the quality of mGovernment service.

### Online information quality

DeLone & McLean (1992) defined the concept of IQ in an online context as “users’ perception of the quality of information presented on a web site”, while Wang & Lin (2012) defined IQ as “the ability of the system to convey the intended meaning of information”. Other studies use the term “content quality” to refer to the concept of “information quality” (Handayani et al., 2018; Kao et al., 2018; Wang, Ou & Chen, 2019). Ensuring the quality of information through online services is essential in maintaining user satisfaction. IQ is the feature of system output that reflects the level of processing data. The fundamental criteria for constructing IQ are relevance, understandability, freedom from error, conciseness, and usability (Lee-Geiller & Lee, 2019). IQ in an online context enables users to make their own decisions on products or services. The quality of information is not just a scale, it is a system that affects user satisfaction (Akram et al., 2019; Haneem et al., 2019; Theodosiou et al., 2019). In this regard, Ostovare & Shahraki (2019) and Sharma & Sharma (2019) pointed out that IQ consists of factors that have to be measured under this scope, such as accuracy, understandability, presentation, and usability. Information provided by electronic portals should facilitate users’ understanding to assist users in performing the transactions as required (Rahi, Abd.Ghani & Hafaz Ngah, 2019; Wang et al., 2019).

IQ has been used for a variety of online quality measurement scales such as e-services, e-commerce, and eGovernment (Chen & Tsai, 2019; Li et al., 2018b; Yang et al., 2018). Liu & Arnett (2000) aimed to measure e-commerce website quality and used the quality dimensions of “information and service quality, system use, playfulness, and system design”, while Barnes & Vidgen (2002) proposed a website service quality model called WebQual 4.0 consisting of three main dimensions: service interaction, information quality, and usability. In the field of eGovernment, Kaisara & Pather (2011) proposed a service quality model consisting of “website design, navigation, site aesthetics, information quality, communication, and trust”, while Bhattacharya, Gulla & Gupta (2012) use eight dimensions, which are citizen centricity, usability, technical adequacy, privacy and security, the usefulness of information, transaction transparency, comprehensive information, and interaction.

The increasing popularity of smartphones has encouraged service providers to launch their services *via* mobile applications to reach users more easily and be more flexible for users to get the required information and services. In this case, measuring the IQ of service-based mobile devices caught the attention of researchers and practitioners (Chen, Vogel & Wang, 2016; Kim, Hwang & Zo, 2016; Tarute, Nikou & Gatautis, 2017). Measuring the service quality of mobile devices required unique measurement scales that can fit with the unique features of smart devices such as portability, limited processors, small screen size, and touch screens (Legner, Urbach & Nolte, 2016; Tam & Oliveira, 2016). Gan & Balakrishnan (2017), Kim, Hwang & Zo (2016) and Liu et al. (2017) argue that measuring the IQ of mobile service requires a measurement scale consisting of unique constructs to enable service providers to understand the quality level of each construct, including information quality (Ahmad & Khalid, 2017; Kao et al., 2018). Mobile government service is a service-based mobile service that is used widely and requires more attention to ensure the quality of the information in the services delivered to users, which influences users’ satisfaction. Few studies proposed a service quality framework for mGovernment, such as a study by Al-Hubaishi, Ahmad & Hussain (2017) that offered a framework consisting of six dimensions: interaction quality, environment quality, information quality, system quality, network quality, outcome quality, while a study by Shareef et al. (2014) consisted of four dimensions: authenticity, interactivity, understandability, and security. The main point here is that the dimension of “information quality” was proposed in the framework of the study by Al-Hubaishi, Ahmad & Hussain (2017) and discussed the general constructs for the relevant proposed dimensions, while the IQ was not included in the model proposed in the study by Shareef et al. (2014). Measuring the IQ of mobile services is a major gap that needs to be considered by researchers, and one of the challenges in the field of mobile government services is to ensure the high quality of information (Adjei-Bamfo, Maloreh-Nyamekye & Ahenkan, 2019; Rahi, Abd.Ghani & Hafaz Ngah, 2019).

Based on previous discussions, the current study aims to analyze the IQ in mobile government services by proposing a model with relative constructs to measure IQ on mGovernment portals. Therefore, the following section discusses the proposed model in detail.

## Mobile government information quality model

The current study aims to define the constructs that can be used to evaluate the IQ of mGovernment services. The dimension of IQ is one of the fundamental service quality dimensions used to measure the nature of information processing before it is put out to end-users. Since few studies report directly on the IQ of mGovernment services, the previous literature in other fields such as electronic government, electronic commerce, electronic service, and mobile application services was reviewed to extract the nature of IQ to be developed in the present study. The absence of service quality models for mobile services leads researchers to use existing service quality models such as e-commerce and e-service to evaluate the quality of service-based mobile applications (Sharma & Sharma, 2019; Wang et al., 2019; Yang et al., 2018). Using other field models in the context of mobile services results in difficulties in measuring the mobile service quality since each context has its features and constructs.

The popularity of mobile devices encourages different sectors (*e.g*., education, health, business, and government) to interact creatively with the public. In that vein, researchers are motivated to investigate IQ among such service-based mobile applications. Chen & Tsai (2019) conducted a study on mobile tourism applications in Taiwan to find the impact of IQ on intention to use. The study by Chen & Tsai (2019) uses six dimensions: information quality, system quality, perceived convenience, ease of use, usefulness, and intention to use. To evaluate the quality of the information in the mobile application, the study uses “accurate, credible, complete, informative, quickly, instantly, and satisfied”. Using the Technology Acceptance Model (TAM) and the Information System Success Model (ISSM), the authors proposed a research model called PLMTA to measure the intention to use mobile applications. Data were collected using an online questionnaire with a total of 213 respondents, of whom 176 respondents were valid. Results show that the IQ dimension affects the intention to use the mentioned mobile applications.

A study of mobile banking was conducted by Sharma & Sharma (2019) to find the quality impact on mobile banking application service usage in Oman. Based on the theory of the D&M IS success model, the authors constructed a model that included the dimensions “information quality, service quality, system quality, trust, satisfaction, intention to use” to measure the actual usage of the mobile application. In addition, the dimension of IQ was evaluated according to the constructs of “up-to-date, easy to understand, and complete”. Results show that IQ influences user satisfaction with the mobile banking application.

A study by Chen et al. (2018) aimed to examine mobile health applications on users’ continued intentions in the health sector. The study used two main dimensions to test the continued intention of users. The first dimension is “perceived usefulness”, consisting of the constructs of “service quality, and information quality”, while the second dimension is “trust”, which consisted of the constructs of “app’s reputation, app’s institution assurance, and privacy concern”. The data were collected using a questionnaire with a sample size of 300 and 284 valid responses. Results show that IQ positively influences the continued use of a mobile application. Another study conducted in the health sector by Kao et al. (2018) aimed to measure mobile health application usability. Kao et al. (2018) used the dimensions of “system usefulness, ease of learning, information quality, interface quality, and overall satisfaction”. The dimension of IQ was constructed according to feedback presented to online users such as “online help, onscreen message, and documentation”. The other constructs used to evaluate IQ were “easy to understand, effectively help users’ complete tasks, and organized”. Using design science research methodology, the authors conducted the evaluation process, and the results show that IQ influences the usability of mobile health applications.

Based on previous literature reviews (see Table 1), the present study extracted IQ constructs that could be used to evaluate the IQ of mobile government service portals.

**Table 1:** Summary of literature reviews.

Row	Author	Environment	Method/Methodology	Dependent variable	Independent variables	IQ constructs	Country
1	Kao et al. (2018)	Mobile health applications	Design science research methodology	Usability	System usefulness, ease of learning, information quality, interface quality, and overall satisfaction	Online help, onscreen message, documentation, easy to understand, effectively help user to complete tasks, and organized	Taiwan
2	Handayani et al. (2018)	Mobile health applications	Questionnaire (127) and Interviews (3)	Success	System quality, information quality, service quality, organization	Easy access, real-time, sufficient and relevant, easy to obtain, easy to read, accuracy, usefulness, and updated	Indonesia
3	Chen & Tsai (2019)	Mobile Tourism Application	Questionnaire (213)	Intention to use	Information quality, System quality, Perceived convenience, Perceived ease of use, Perceived usefulness, Intention to use	accurate, credible, complete, informative, quickly, instantly, and satisfied	Taiwan
4	Sharma et al. (2018)	Mobile Government Application	Questionnaire (400)	Behavioral intention	Performance Expectancy, Effort Expectancy, Social Influence, Facilitating Conditions, Trust, Information quality, Behavioral Intention	Up-to-date, and complete	Oman
5	Tarute, Nikou & Gatautis (2017)	Mobile Business Application	Questionnaire (246)	Continuous use	Perceived functionality, Design, Information quality, Interaction (consumer & content), Consumer engagement, Continued intention to use	Informative of application, availability of various information, and engaging	Lithuania
6	Chi (2018)	Mobile commerce	Questionnaire (786)	Intention to Use	Brand Loyalty, Brand Association, Perceived Quality, Brand Image, Information Quality, System Quality, Service Quality	Up-to-date, accurate, comprehensive, attractive, attention, and informative	China
7	Wang et al. (2019)	Mobile catering	Questionnaire (196)	Success	System quality, information quality, service quality, product quality, perceived price, perceived promotions, perceived value, user satisfaction, intention to reuse, and eWOM	Precise, sufficient, up-to-date	Taiwan
8	Sharma & Sharma (2019)	Mobile Banking	Questionnaire (227)	Actual usage	Information quality, Service quality, System quality, Trust, Satisfaction, Intention to use, Actual usage	Up-to-date, understandable, and complete	Oman
9	Gao, Waechter & Bai (2015)	Mobile commerce	Questionnaire (462)	Continued intention towards mobile purchase	System quality, Information quality, Service quality, Privacy and security concerns, Trust, Flow, Satisfaction, Continued intention	Relevant, sufficient, accurate, and up-to-date	China
10	Chen et al. (2018)	Mobile health applications	Questionnaire (284)	Continuance intention	Perceived Usefulness: [service quality, information quality], Trust: [App’s reputation, App’s institution assurance], Privacy concern	Accurate, adequacy, timeliness	China
11	Legner, Urbach & Nolte (2016)	Mobile Business Application	Questionnaire (374)	Mobile applications’ design, success	System quality, information quality, process quality, service quality, use, User satisfaction, Individual benefits, Management support	usefulness, understandability, and timeliness	China
12	Gan & Balakrishnan (2017)	Interactive Mobile Messaging App	Pretest survey (38) students	Mobile Interaction	Perceived usefulness, Perceived ease of use, Self-efficacy, Enjoyment, Uncertainty avoidance, System quality, Information quality, Adoption intention	Presented way, understandability, attractive, and organized	Malaysia
13	Tam & Oliveira (2016)	Mobile Banking	Questionnaire (233)	user satisfaction	System quality, Information quality, Service quality	Useful, understandable, interesting, reliable, complete, and up-to-date	Portugal
14	Tseng & Lee (2018)	Mobile commerce	Questionnaire (303)	System characteristics	System quality, information quality	Relevant, easy, and accurate	Global
15	Kim, Hwang & Zo (2016)	augmented reality	Questionnaire (1,200)	Continuous Intention	Information Quality, Interactivity, Visual Quality, Perceived Usefulness, Perceived Enjoyment, Satisfaction	Needed, up-to-date, clear and understand	Korea

### Understandability IQ

The concept of understandability refers to the degree of information that can be easily understood by the user/reader (Dikici, Turetken & Demirors, 2018; Ojha, Ismail & Kuppusamy, 2018). It evaluates the user’s efforts to understand the information as targeted (Eom & Kim, 2014; Lorenzi et al., 2014). It reflects the user's learning process that constructs a knowledge guided to perform the task (Ahmad & Khalid, 2017; Aloudat et al., 2014). Understandability of online platforms such as electronic services and mobile services is affected by factors such as the complexity of information, contents, focus, and givenness (Liu et al., 2014; Wang, 2014). It has been used in various sectors to evaluate service-based mobile applications. In the health sector, studies by Chen et al. (2018), Handayani et al. (2018), and Kao et al. (2018) used the construct of “understandability” to refer to mobile service IQ in the banking sector (Sharma & Sharma, 2019; Tam & Oliveira, 2016), and in the business and commerce sectors (Legner, Urbach & Nolte, 2016; Tseng & Lee, 2018). IQ in mobile services is related to the unique features of mobile services, such as mobility, small screens, and limited processing compared with desktop devices. Based on the previous literature, the construct of “understandability” leads to enhanced quality of information of service-based mobile applications, which is essential nowadays to be evaluated on mGovernment portals.

### Timeliness IQ

Timeliness IQ has been used in the field of information systems to describe the process of delivering the data quality on time to meet user demand (Rahi & Abd.Ghani, 2019; Roy, Sreejesh & Bhatia, 2019). James & Sammy (1983) defined timeliness as “the availability of the output information at a time suitable for its use”, while Wang & Strong (1996) defined it as the extent to which the data is available to use at the required time. The service provider’s refreshment process may affect receiving data on time by end-users. Therefore, it can influence user satisfaction (Resende & Cardoso, 2019; Zuo et al., 2019). Thus, the availability of information on the electronic portal must be ensured to assist users in performing the required tasks. Timeliness IQ has been measured in mobile application services. Handayani et al. (2018) conducted a study in the health sector and used the term “updated” to measure the availability of health information in mobile application services. Handayani et al. (2018) argue that updates of information help users act on time. In the field of mobile commerce, Chi (2018) evaluated the information based on timeliness and found that it influences the adoption of such services by consumers. It has been used in previous studies on various mobile service applications to evaluate IQ such as Gao, Waechter & Bai (2015), Kim, Hwang & Zo (2016), Sharma et al. (2018), Sharma & Sharma (2019), Tam & Oliveira (2016), and Wang et al. (2019). Based on the previous literature, it can be concluded that measuring the timeliness of mGovernment services is important to help users get the required information that guides them to take decisions and perform transactions on time.

### Accuracy IQ

The accuracy of information is formulated as output to users of an online platform. It has been explained as the overall objective percentage that reflects data errors and out-of-range values (Naumann & Rolker, 2000). Redman (1996) defines it as the “degree of agreement between a collection of data values and a source agreed to be correct”. The accuracy of online information provides insight (Theodosiou et al., 2019; Tuzkaya et al., 2019). One of the main reasons for the provision of electronic services is to provide accurate information to users. This means that the user expects correct and precise information (Gracia-Tinedo et al., 2018; Li et al., 2018a). Studies of mobile service quality have included the concept of accuracy as a construct to evaluate the dimension of IQ. Chen et al. (2018), Gao, Waechter & Bai (2015), Tseng & Lee (2018), and Wang et al. (2019) have used the term “accuracy” to evaluate mobile services’ IQ. However, the previous literature shows that accuracy can enhance the evaluation process applied to IQ in terms of correct information that is free of errors, subtle, and targets the service range.

### Completeness IQ

The concept of completeness of information describes the integration of all the necessary parts belonging to the information to construct knowledge, assisting end-users to decide (Almarashdeh & Alsmadi, 2017; Jia, Yang & Jiang, 2018). This includes whether the information is required to be completed to provide minimum knowledge that helps people make accurate decisions. In online services, it is also necessary to give complete information to the users, especially when the transaction must be done with the support of another source of information. One of the fundamental goals of electronic services is to provide information and services available to the public; hence the service provider must ensure and guarantee that the electronic portal contains complete information. The concept of completeness as an essential construct of IQ has been used in previous studies on mobile services such as Chen & Tsai (2019), Chi (2018), Kao et al. (2018), Sharma et al. (2018), Sharma & Sharma (2019), and Tam & Oliveira (2016). Therefore, the present study proposes the concept of accuracy as a construct to evaluate the IQ of mobile government services.

### Availability IQ

The concept of availability of IQ refers to the ability of the infrastructure to perform the required functions according to the expectations of end-users within a specified time of operation. It guarantees the accessibility of information whenever requested in the correct format. When the system is non-functioning, it affects the availability of information and the impact on the users’ satisfaction. Shayestehfar & Yazdani (2019), Verma, Chaurasia & Bhattacharyya (2019), and Woo (2019) stated that the availability of information is related to the security policies of the system or service provider. This means that the security is managed by the status or conditions of the availability of information in an online environment. Different studies such as Chen & Tsai (2019), Handayani et al. (2018), Kao et al. (2018), and Tarute, Nikou & Gatautis (2017) have measured it in various sectors of mobile services. Based on previous studies, it is essential to include the construct of “availability” that enhances IQ evaluation for mobile government services.

### Usefulness IQ

**Figure 1:**
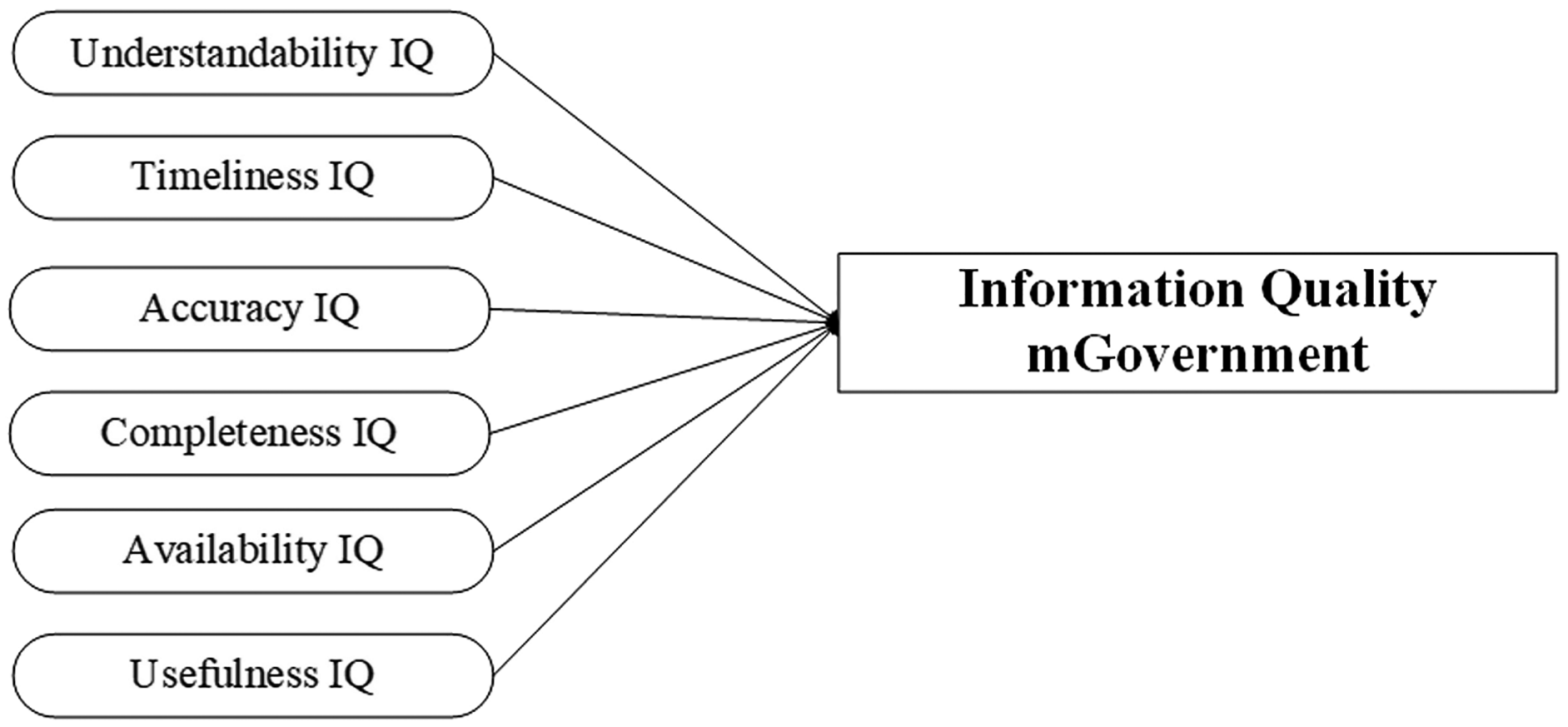
Proposed model of information quality at mGovernment service.

The construct of information usefulness refers to the extent to which the information is perceived as valuable by the users/readers. It is a construct that influences end-users’ satisfaction with the online environment. It refers to the status of information that can be helpful, beneficial, and makes sense in people’s lives. The construct of “usefulness IQ” has been used by previous studies as a construct to evaluate the dimension of information, such as Handayani et al. (2018), who use it to evaluate the quality of mobile health applications, and Legner, Urbach & Nolte (2016), who use it to evaluate the quality of mobile business application, while Tam & Oliveira (2016) use it to evaluate the quality of mobile banking application, and Kim, Hwang & Zo (2016) use it as a construct of information to evaluate the mobile augmented reality application. Using the construct of usefulness in online services can manage the content of information to illustrate the contents with direct attention to the users. On the other hand, the issues of non-usefulness of information can exist in the extensive contents of the information in a specific area such as a website page, which negatively impacts users’ attention and requires more time to take in the key point of its usefulness. In the case of mGovernment services, the unique features are the small screen and limited functions, which require providing essential and useful information in the mobile application to assist users to perform the tasks and save time. Therefore, the construct of the usefulness of information must be measured in the context of mobile government services with consideration of the unique features of mobile devices as illustrated in Fig. 1.

## Research methodology

### Search methods

**Figure 2:**
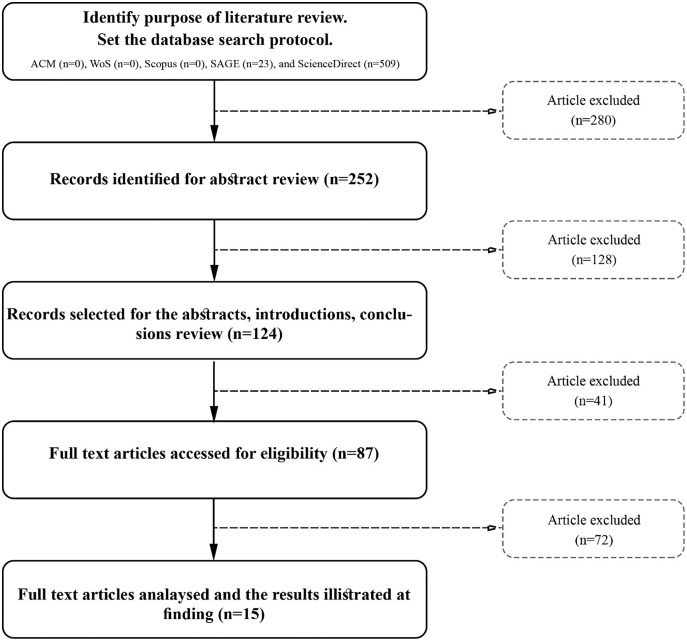
Flow chart of the systematic literature review.

This article follows the search methods discussed by Okoli (2015), which guide the performance of the research stages of planning, selection, extraction, and execution of literature reviews as shown in Fig. 2.

### Database libraries search

The search was conducted on five databases: ACM, WoS, Scopus, SAGE, and ScienceDirect, covering the period between 2014 and 2019. The search keywords were selected according to the context of the current article, and the search was performed based on the complex of the following keywords: “information”, “information quality”, “online information quality”, “electronic information quality”, “mobile information quality”, “system information quality”, “mobile government”, “mGovernment”, “mobile government service”, “mGovernment service”, “mobile” and “mobile service”. In addition, the Boolean operators of “AND” and “OR” were used among the search keywords.

The search procedure conducted, consisting of five main stages, is illustrated in Fig. 2. At the planning stage, the researcher identifies the purpose of the literature review. Then, at the selection stage, the researcher performs the search of literature with the practical screen. The main criteria here are to find the latest studies during the period 2014–2019, review and research articles, and the context of the study on mobile services. The result shows 532 articles. Conducting screening on this result ensures that the study is done in the context of any service-based mobile application and that the study aims to measure, evaluate, and propose a model of quality with consideration of the dimension of IQ. Hence the result is 15 articles that meet the targeted criteria of the present research (see Tables 1 and 2).

**Table 2:** The constructs used by previous literature to evaluate information quality at service based mobile.

Row	References and categories of IQ constructs	Easy/under-standable	Up-to-date	Accurate	Complete	Organized	Usefulness	Timeliness	Attractive	Sufficient	Relevant	Informative	Accessible	Available	Reliable	Adequacy/satisfied/interested	Engaging/attention/presented way	Total
1	Kao et al. (2018)	✓			✓	✓							✓	✓				5
2	Handayani et al. (2018)	✓	✓	✓			✓			✓	✓		✓	✓				8
3	Chen & Tsai (2019)			✓	✓							✓	✓	✓	✓	✓		7
4	Sharma et al. (2018)		✓		✓													2
5	Tarute, Nikou & Gatautis (2017)											✓		✓			✓	3
6	Chi (2018)		✓	✓	✓				✓			✓					✓	6
7	Wang et al. (2019)		✓	✓						✓								3
8	Sharma & Sharma (2019)	✓	✓		✓													3
9	Gao, Waechter & Bai (2015)		✓	✓						✓	✓							4
10	Chen et al. (2018)			✓				✓								✓		3
11	Legner, Urbach & Nolte (2016)	✓					✓	✓										3
12	Gan & Balakrishnan (2017)	✓				✓			✓								✓	4
13	Tam & Oliveira (2016)	✓	✓		✓		✓								✓	✓		6
14	Tseng & Lee (2018)	✓		✓							✓							3
15	Kim, Hwang & Zo (2016)	✓	✓				✓											3
Total		8	8	7	6	2	4	2	2	3	3	3	3	4	2	3	3	

The execution stage enables the quality appraisal and extraction of the data. The extraction of the article’s data is tabulated with the following: row number, author, environment/context, method/methodology, dependent variable, independent variables, IQ constructs, and country. The final stage is execution, which enables the authors to perform the analysis according to the findings and write the literature review.

### Ethical consideration

The ethical considerations considered while conducting the study include ensuring the accuracy and fairness of articles. The library databases used are within the official list of the University of Malaya.

## Research implications

The study proposed a model that can evaluate IQ in mobile government services. It enables government agencies to get an in-depth analysis and understanding of users’ expectations toward the delivery of services in terms of IQ. Measuring and developing the service from the perspective of information can influence user satisfaction and impacts the continued use of such services. The constructs of the proposed model have been analyzed and discussed to ensure their suitability to be parts of the IQ model. This helps researchers to test the model as qualitative or quantitative studies, while it enhances the practitioners to apply it to any services on the mGovernment portal to improve the service to meet users’ satisfaction.

## Research limitations

The study has been conducted within the scope of services provided in the form of mobile applications by government agencies using mobile devices. Therefore, the study is limited to investigating the IQ factor considering its unique features when applied to mobile devices. However, further research is needed to conduct more studies on the other service quality dimensions of mGovernment such as “usability, interaction, security and privacy”.

## Conclusions

Ensuring quality in any field is the key to success. With the development and extension of technologies in service delivery, an effort must be made by researchers for more analysis and investigations. However, mobile government is still in the development process, and countries are transferring their services from the eGovernment to the mGovernment portal. Measuring the quality of service of mGovernment is necessary to identify the users’ needs and expectations. Therefore, a compatible model should measure and evaluate the service quality delivered to users by mGovernment applications. IQ is a fundamental dimension, and this encouraged the authors of the current study to conduct more analysis to investigate the related constructs that can measure the quality of information on the mGovernment portal. The study proposes a model of IQ consisting of “understandability, timeliness, accuracy, completeness, availability, and usefulness”. The study provides a guide for understanding the quality delivered through mobile devices and the impact of IQ on these services.

## Additional Information and Declarations

### Data Availability

The following information was supplied regarding data availability: The raw data is available in the Tables.
